# Patients’ Perspectives on Breast Reconstruction in Sub-Saharan Africa

**DOI:** 10.1001/jamanetworkopen.2025.17749

**Published:** 2025-06-26

**Authors:** Natalie M. Guzman, Brigit D. Baglien, Eden S. Kassa, Ishmael Kyei, Paa Ekow Hoyte-Williams, Mahteme Bekele Muleta, Sarah T. Hawley, Mary E. Byrnes, Adeyiza O. Momoh

**Affiliations:** 1 Department of Learning Health Sciences, University of Michigan, Ann Arbor; 2 Center for Healthcare Outcomes and Policy, University of Michigan, Ann Arbor; 3 Section of Plastic and Reconstructive Surgery, Department of Surgery, University of Michigan, Ann Arbor; 4 Department of Surgery, St Paul’s Hospital Millennium Medical College, Addis Ababa, Ethiopia; 5 Department of Surgery, Komfo Anokye Teaching Hospital, Kumasi, Ghana; 6 Department of Internal Medicine, University of Michigan, Ann Arbor; 7 Department of Health Behavior and Health Education, University of Michigan, Ann Arbor; 8 Ann Arbor VA Center for Clinical Management Research, Ann Arbor, Michigan; 9 Department of Surgery, University of Michigan, Ann Arbor

## Abstract

**Importance:**

Mastectomy hesitation among women with breast cancer in sub-Saharan Africa contributes to late presentation, advanced disease, and high mortality rates in the region. Mastectomy alone has been found to substantially reduce quality of life among sub-Saharan African women. Breast reconstruction may improve quality-of-life sequelae from mastectomy, as well as improve the acceptance of oncologic surgical treatment. Little is known about women’s attitudes and perceptions about breast reconstruction in the African context.

**Objective:**

To explore sociocultural perspectives, attitudes, and perceptions on breast reconstruction among female patients with breast cancer in sub-Saharan Africa.

**Design, Setting, and Participants:**

This qualitative study of patients with breast cancer was conducted in December 2023 and February 2024. Content analysis was performed from February 2 to June 27, 2024. Two large urban academic hospitals, Komfo Anokye Teaching Hospital and St Paul’s Hospital Millennium Medical College, identified eligible patients during visits to breast cancer clinics.

**Main Outcomes and Measures:**

Perspectives, attitudes, and perceptions on breast reconstruction were obtained using semi-structured interviews with patients with breast cancer.

**Results:**

A total of 46 women were interviewed, 25 in Ghana (mean [SD] age, 46.1 [8.9] years) and 21 in Ethiopia (mean [SD] age, 48.7 [8.3] years). Three major themes were identified. First, women’s perceptions toward breast reconstruction were shaped by a balance of breasts contributing to self-identify vs breasts serving only a functional purpose. Second, attitudes were influenced by the life stage of a woman, such that reconstruction was viewed as beneficial for younger, dating women, but benefits decreased as women age. Third, opinions on breast reconstruction varied between perceiving reconstruction as natural or as contradicting divine will.

**Conclusions and Relevance:**

In this qualitative study of women with breast cancer in Ghana and Ethiopia, there was a complex interplay of factors associated with shaping these women’s perspectives toward breast reconstruction. These findings provide insights that may aid in the development of tailored interventions specific to women in sub-Saharan Africa. Such interventions may have implications for improved patient-centered counseling, quality of life, and holistic breast cancer care.

## Key Points

**Question:**

What are sub-Saharan African women’s perspectives on postmastectomy breast reconstruction?

**Findings:**

In this qualitative study of 46 women with breast cancer from Ghana and Ethiopia, 3 major themes influenced opinions toward breast reconstruction: the balance between self-identity and functionality, life-stage considerations (ie, benefits decrease with age), and views on divine will and naturalness. Breast reconstruction was seen as valuable for restoring confidence and symmetry but conflicted with cultural and religious beliefs for some participants.

**Meaning:**

These findings suggest that efforts to align expansion of breast reconstruction programs in sub-Saharan Africa with patients’ values should consider sociocultural, life-stage, and religious factors.

## Teaser

This qualitative study explores sociocultural perspectives, attitudes, and perceptions about breast reconstruction among women with breast cancer in Ghana and Ethiopia.

## Introduction

Breast cancer is the leading cause of cancer-related deaths in sub-Saharan Africa, with a 5-year survival rate of only 40% compared with 85% to 95% in high-income countries.^1,2,3,4^ This disparity is due to multiple factors, including insufficient screening, oncology specialist shortages, limited drug access, and delayed diagnosis presenting as advanced-stage disease.^5,6,7,8^ In Ghana and Ethiopia, late-stage presentation has been identified as a primary factor for poor prognosis.^7,8^ Mastectomy remains the primary surgical treatment due to late presentation and limited adjuvant therapy access.^9^

As breasts are considered an attribute of femininity, maternity, and sexuality, losing a breast can affect a woman’s quality of life.^9^ Prior work in Ethiopia found that women who underwent mastectomy reported a lower quality of life compared with international findings.^9^ A recent study also found that mastectomy was associated with a reduced satisfaction with breasts and sexual well-being in a cohort of Ethiopian and Ghanaian women.^10^ In high-income countries, breast reconstruction after mastectomy provides well-documented benefits for cancer survivors, including improved psychological well-being, body image, and breast satisfaction.^11,12,13,14,15,16,17^ Despite expanding access in sub-Saharan Africa, breast reconstruction remains uncommon after mastectomy, and its role in African health care contexts is poorly understood.^18,19^ It is unclear whether sub-Saharan African women would benefit from reconstruction similarly to women in high-income countries and whether reconstruction could support a more holistic approach to care.

Thus, this study explored the sociocultural perspectives on breast reconstruction among patients with breast cancer in Ghana and Ethiopia, 2 countries in sub-Saharan Africa with a growing body of literature that our study could build on. While reconstruction access is currently limited, plastic surgery capacity is growing in these countries.^20,21^ Understanding patient perspectives and cultural context is essential before implementing and expanding breast reconstruction programs.^22^ Findings from this study may inform future strategies tailored to African women for the possible expansion of breast reconstruction to the existing multidisciplinary breast cancer care efforts in the region.

## Methods

In this qualitative study, our findings are in the context of a larger, mixed-methods study, the Sub-Saharan Africa Breast Reconstruction study. We collected both qualitative and quantitative data that examined mastectomy concerns, reconstruction awareness, and access barriers among sub-Saharan African patients with breast cancer. This report focuses on emergent findings from our qualitative interviews. We use results from the convergence of our mixed-methods study to inform the development of a culturally tailored breast reconstruction decision aid.

The University of Michigan’s Institutional Review Board deemed this study as low risk to participants (ie, identity not easily ascertained, disclosure of responses would not reasonably place participants at risk) and exempt from ongoing review. Institutional ethics review committees of Komfo Anokye Teaching Hospital in Ghana and St Paul’s Hospital Millennium Medical College in Ethiopia provided approval for this work. All participants provided written informed consent upon arrival to interviews. We followed the Standards for Reporting Qualitative Research (SRQR) reporting guideline.

### Study Design and Participants

Our study design used interpretive description, a qualitative methodology aimed at addressing real-life clinical problems.^23^ Interpretive description uncovers meanings in qualitative data that center the participant voice and experiences while maintaining clinical relevance.^23,24^ We leaned on the practicality of interpretive description to provide rich and context-driven insight.

Participants were recruited through convenience sampling at breast cancer clinics at Komfo Anokye Teaching Hospital and St Paul’s Hospital Millennium Medical College under the supervision of site investigators (I.K. and P.E.H.-W. in Ghana and M.B. in Ethiopia). Women aged 18 to 70 years diagnosed with breast cancer, either before or after mastectomy (stage 0-3) who spoke English, Amharic, or Twi were eligible for our study. Study coordinators contacted eligible patients seen within the previous year and invited those able to attend in person. Participants received US $20 compensation.

We determined our sample based on information power, which considers sample specificity, breadth of study aim, quality of dialogue, and analytic strategy.^25^ Similar qualitative studies included 18 to 35 participants.^26,27^ However, despite our narrow study aim and team-based consensus analytic approach, which may precipitate a smaller sample size, we were less certain about dialogue quality due to language and cultural barriers. Therefore, we aimed for a larger sample than may have been necessary.

### Interview Procedures

Semistructured interviews were conducted during 1-week periods in December 2023 in Ghana and February 2024 in Ethiopia. Interviews occurred in private clinic or conference rooms with the participant, a local study team member serving as translator, and a US study team member. The interviews were conducted by women on the study team (N.M.G., B.D.B., and M.E.B.) who were trained in qualitative methods and not involved in delivering patient care. Questions were first asked in English, then translated into the participant’s native language, and then translated back in English. Interview duration ranged from 20 to 65 minutes.

Audio recorders were used to capture interviews, with audio files subsequently uploaded to secure devices. A transcription service converted audio files into text. N.M.G. and B.D.B. then read line by line while listening to the transcript to verify accuracy and remove identifying information and non-English portions. Our interview guide focused on the woman’s background, her process of diagnosis and treatment, the meaning of losing 1 breast or both breasts, impacts on quality of life and well-being, and opinions and knowledge related to breast reconstruction (eMethods in Supplement 1).

The interview guide was initially informed by the World Health Organization’s Conceptual Framework for Action on the Social Determinants of Health and was iteratively revised to focus on cultural and societal values, as well as socioeconomic issues.^28^ We collaborated closely with local physician partners in Ghana and Ethiopia, leveraging their understanding of patient experiences to strengthen our interview approach.

### Techniques to Enhance Trustworthiness and Reflexivity

We triangulated data through 3 methods: (1) team debriefings after interviews to verify interpretations with local physicians, (2) researcher triangulation with multiple team members collecting and analyzing data, and (3) continuous reference to the literature throughout our analytic process. We acknowledged our positionality as a study team of White western women conducting interviews and performing analysis. Our identities may have influenced responses, and our limited cultural understanding of Ghana and Ethiopia may have constrained our analysis, making our collaboration with local partners essential. Coming from our privileged perspectives, we could not fully comprehend barriers to breast cancer treatment faced by women in these countries. Despite these limitations, we prioritized interpreting data from the perspective of the women who shared their stories and highlighting their voices.

### Data Analysis

Between February 2 and June 27, 2024, data were analyzed iteratively using a team-based approach informed by interpretive description principles.^29^ Transcribed interviews were uploaded into MAXQDA, version 24 (VERBI GmbH) for qualitative analysis. Three team members (N.M.G., B.D.B., and M.E.B.) independently reviewed every transcript to identify initial codes and develop a codebook, which was further revised in subsequent meetings until verbal consensus was reached (eTable in Supplement 1). Two team members (N.M.G. and B.D.B.) then independently coded the transcripts and wrote memos to understand the data at a deeper level. Coding was conducted from the bottom up, generating codes from the data rather than applying a preexisting theoretical framework.^30,31^

After coding, the team created a matrix, a visual framework organizing qualitative data in tabular format, to examine associations between codes and drafted theme reports and revised these in weekly meetings.^32^ Key categories were compared to identify similarities and were then organized into major themes and subthemes, which served as a structure to report our qualitative findings.^33^ Throughout the process, we engaged in extensive discussions with our local partners to ensure that the identified themes aligned with their observations of patient experiences.

## Results

We interviewed a total of 46 women, 25 in Ghana (mean [SD] age, 46.1 [8.9] years) and 21 in Ethiopia (mean [SD] age, 48.7 [8.3] years). Patient demographics are outlined in Table 1. Most (41 [90%]) had undergone mastectomy, and none underwent reconstruction. These women’s opinions toward breast reconstruction were shaped by 3 main themes: (1) balancing self-identity and functionality, in which women who valued breasts for identity favored reconstruction, while those focused on functionality viewed it as an unnecessary burden; (2) life stage, in which reconstruction was viewed as beneficial for younger, dating women, but benefits decreased as women age; and (3) divine will and naturalness, in which some saw reconstruction as opposing God’s will, while others viewed it as natural breast restoration.

**Table 1.** Participant Characteristics

Characteristic	Participants, No. (%)	
	Ghana (n = 25)	Ethiopia (n = 21)
**Sociodemographic characteristics**		
Age, mean (SD), y	46.1 (8.9)	48.7 (8.3)
Relationship status		
Married	6 (24)	13 (62)
Divorced or separated	2 (8)	3 (14)
Widowed	1 (4)	4 (19)
Single	8 (32)	1 (5)
Missing response	8 (32)	0
Employment status		
Employed	7 (28)	6 (29)
Unemployed	10 (40)	7 (33)
Retired	0	4 (19)
Homemaker	0	4 (19)
Missing response	8 (32)	0
Religion		
Christian	16 (64)	18 (86)
Muslim	0	3 (14)
Missing response	9 (36)	0
Insurance		
Yes	12 (48)	16 (76)
No	0	4 (19)
Missing response	13 (52)	1 (5)
Education level		
None	2 (8)	0
Primary school	4 (16)	3 (14)
Middle school	6 (24)	3 (14)
High school	2 (8)	6 (29)
College (>2 y)	0 (0)	8 (38)
Graduate school	3 (12)	1 (5)
Missing response	8 (32)	0
**Health-related characteristics**		
Diabetes		
Yes	1 (4)	2 (10)
No	16 (64)	19 (90)
Missing response	8 (32)	0
Smoking		
Yes	1 (4)	0
No	16 (64)	21 (100)
Missing response	8 (32)	0
Breast cancer type		
Ductal carcinoma in situ	1 (4)	0
Invasive ductal carcinoma	14 (56)	12 (57)
Lobular carcinoma in situ	1 (4)	7 (33)
Other	1 (4)	2 (10)
Missing response	8 (32)	0
Chemotherapy		
Neoadjuvant	11 (44)	2 (9)
Adjuvant	1 (4)	15 (68)
None	2 (8)	5 (23)
Missing response	11 (44)	0
Radiation therapy		
Yes	7 (28)	3 (14)
No	6 (24)	14 (67)
Missing response	12 (48)	4 (19)

### Balancing Self-Identity and Functionality

#### Restoration of Confidence and Feelings of Wholeness

Women favoring reconstruction explained that it could restore physical attributes lost during mastectomy, potentially improving self-esteem and well-being (Table 2). A Ghanian woman in her 40s stated that “[reconstruction] will boost my self-confidence, my image, and make me feel good” (participant G25). In both countries, women cited improved self-confidence as one of the main benefits of reconstruction.

**Table 2.** Balancing Self-Identity and Functionality

Subtheme	Example quotes (participant)
Reconstruction restores confidence and feelings of wholeness	“If I can get another one to make it two again, [I like] it.” (Ghanian woman in her 40s [participant G15]) “What would push me to do it is that when [the doctors] do the mastectomy, I lose one of my breasts. When I’m wearing clothes, I will not be comfortable, [I] will not look attractive. So, once I am able to get another breast fold, [it will] help me feel more confident, I’ll look more attractive in the clothes.” (Ghanian woman in her 30s [participant G22]) “Having a reconstructive breast would help in having more confidence [in the] appearance of my body, and not having [a reconstructive breast] might cause [me] low self-esteem.” (Ethiopian woman in her 30s [participant E6]) “Some women get broken so easily, and especially when the disease [is a] burden, [then] losing the breast is an additional burden and if [breast reconstruction] is provided for them by the time they have mastectomy, it helps [women] avoid the psychological impact they are going to have after the surgery. I think it’s helpful.” (Ethiopian woman in her 50s [participant E19])
Reconstruction and the role of utility	“I’m not so particular about getting a new breast, but I will think about it…because I don’t need a breast for anything for now…it is not a priority.” (Ghanian woman in her 20s [participant G3]) “[I don’t] want it. [I don’t] think that it helps much. [My] understanding is to just have the tumor removed and to heal.” (Ethiopian woman in her 30s [participant E9]) “[Breast reconstruction] is just for the physical [appearance], and otherwise I don’t [need it]. I am not going to breastfeed from that breast, so it’s just for the look and appearance.” (Ethiopian woman in her 50s [participant E18]) “I think both options [breast prosthesis and breast reconstruction] are not good options for me. It doesn’t change [anything] or prolong your life on Earth.” (Ethiopian woman in her 50s [participant E21])

Some women expressed disappointment with postmastectomy asymmetry and valued reconstruction for restoring symmetry to their body and feelings of wholeness. A Ghanian woman in her 30s stated, “[Breast reconstruction] is good because you get your breast back and you feel good that the rest [of your body] is there” (participant G27). Women also noted that reconstruction may help reverse some of the negative psychological outcomes associated with mastectomy (Table 2).

#### Reconstruction and the Role of Utility

In contrast, other women described reconstruction as an additional burden and did not believe that their breasts were important contributors to their image or well-being (Table 2). This view often was held by women viewing their breasts from a solely functional perspective. As an Ethiopian woman in her 50s stated, “[I think reconstruction] is not necessary because it’s faking oneself, but it’s like, [the reconstructed breast] is not a breast…it doesn’t do what the breast does…it’s an additional burden” (participant E21). This woman viewed reconstruction as inauthentic since reconstructed breasts lack natural function, making the procedure more burdensome than beneficial. Overall, women who viewed breasts through a utilitarian lens found reconstruction to be unnecessary. While there were positive and negative views in both countries, Ghanian women more commonly viewed reconstruction as important to their healing journey, self-esteem, and self-concept, whereas Ethiopian women more frequently viewed it as unnecessary because it does not restore breast functionality.

### Life Stage

Breast reconstruction was viewed as acceptable for younger women due to the benefits of increased self-confidence and self-esteem. On the other hand, cultural norms tended to be unsupportive of breast reconstruction for older women (Table 3). An Ethiopian woman in her 40s stated that “since I am old, I don’t need it in my opinion…but for younger patients with breast cancer, I think it’s a good option because they don’t even have nice clothes [that fit]. [Her body] will be asymmetric, so I think for them, it’s a good idea” (participant E1). This participant’s point of view highlights the consensus that reconstruction benefits younger women by maintaining attractiveness and breast symmetry, qualities deemed more significant at younger ages. If women considered themselves as older, the benefits of reconstruction were seen as less relevant, underscoring a belief that there is a perceived turning point in age in which older women should not value appearance. For some women, they felt that they had reached this turning point and that their time had passed, such as a Ghanian woman in her 40s who said, “I reached the age that I don’t need to get another breast” (participant G16).

**Table 3.** Life Stage

Subtheme	Example quotes (participant)
Reconstruction benefits are life-stage dependent	“[Breast reconstruction] is good for those younger women or women who prefer to still have their breast. But I am done breastfeeding, and I don’t need my breast for anything. At my age, I don’t need a breast anymore, and if a man truly loves me and wants to be in a relationship, I will tell him the truth about my situation. If the man is willing to stay with me, I have no problem. If he wants to leave, I don’t have any problem with that.” (Ghanian woman in her 60s [participant G11]) “It depends on the person; I would recommend her [a younger women] to get the breast reconstruction so that she would feel good [about herself] and she would feel pretty about herself.” (Ethiopian woman in her 60s [participant E10]) “It’s good for other people, like younger people. But for me, since I’m older and since I’m a widow, I don’t think it’s good.” (Ethiopian woman in her 50s [participant E12]) “I don’t want it. But it is important, especially for the youngsters…it’s good like, whenever you look at yourself through the mirror or in front of your family, if you want to change your clothes or something, having something missing from your body is not a good feeling.” (Ethiopian woman in her 50s [participant E19])

Reconstruction was also seen as advantageous for women who are dating and trying to establish their family. An Ethiopian woman in her 50s stated that reconstruction was only beneficial for “those who are dating and single and looking for a husband” (participant E16). Women often viewed reconstruction as unnecessary after completing childbearing and breastfeeding. While older age was a stronger deterrent in Ethiopia, participants in both countries felt that reconstruction conflicted with cultural values around aging.

### Divine Will and Naturalness

#### Reconstruction as Unnatural and Against Divine Will

Several participants described reconstruction as unnatural and believed it went against God’s will (Table 4). An Ethiopian woman in her 40s stated, “I don’t believe in it. I believe in nature. So, if what was created there was removed, then I would rather [it] stay that way than having something unnatural placed” (participant E8). Additionally, other women believed it was part of God’s plan to have their breast removed, as an Ethiopian woman in her 60s explained, “so once my breast is gone, I don’t think it’s necessary to have an operation, I think it is God’s plan” (participant E2). While this woman accepted mastectomy as God’s plan, reconstruction was viewed differently and was not considered to be a part of what God intended for her.

**Table 4.** Divine Will and Naturalness

Subtheme	Example quotes (participant)
Reconstruction is unnatural and against divine will	“No, it’s not good….[I] will not actually opt for it…that what God has given you naturally, you can’t compare to what other people will do for you.” (Ghanian woman in her 40s [participant G21]) “[I am not satisfied with the asymmetry], but I still do not want to go through [breast reconstruction]. I have already accepted that even though I don’t like it, I’m trying to accept that and thank God, I’m healthy, that’s all that matters. I have used my breasts, and all my kids are now grown. I don’t complain.” (Ethiopian woman in her 50s [participant E12])
Reconstruction is authentic and natural	“[I] would have loved to go through reconstruction and get [my] breast, natural breast back.” (Ghanian woman in her 60s [participant G5]) “You look natural after reconstruction, and you do not look deformed. The breast is not dead, so it’s good.” (Ghanian woman in her 40s [participant G25])

Women opposed reconstruction for various religious reasons, including viewing it as an artificial solution not aligning with religious norms, believing that they should focus on gratitude for survival rather than mastectomy sequalae. Some even considered mastectomy and reconstruction as sinful, as an Ethiopian woman in her 40s stated, “[Mastectomy] is like some sort of a sin because you are reducing the part of the body that God has given you. So, on top of that, doing another surgery to create other breasts, it’s not acceptable for me” (participant E15).

#### Reconstruction as Authentic and Natural

In contrast, other women found reconstruction to be an authentic process that aligned with their beliefs (Table 4). Many of these women viewed breast reconstruction through a body-positivity lens and reconstruction itself as natural, as a Ghanian woman in her 50s stated, “I only know of prosthesis, but it’s wonderful to see someone who has had bilateral breast mastectomy having new breasts, like [the breasts] being constructed for that person. To see someone with a new breast, a natural breast, it’s a wonderful thing” (participant G6).

Views on reconstruction as either natural or contradictory to religious beliefs had pronounced influences on participants’ opinions. Ghanaian women commonly viewed reconstruction positively as a natural process, while Ethiopian women frequently cited reconstruction as conflicting with their religion and beliefs. While this view was more common in Ethiopia, there were still several participants in Ghana who considered reconstruction as contradictory to their religion and beliefs.

## Discussion

This qualitative study examined attitudes and perceptions toward breast reconstruction among patients with breast cancer in Ghana and Ethiopia. Three key themes emerged: the balance between identity and functionality, the influence of life stage, and views on divine will vs natural restoration. Women valuing breasts for body image favored reconstruction, while others saw it as unnecessary surgery. Reconstruction was considered more beneficial for younger women’s self-esteem and family planning. Religious views varied, with some seeing it as restorative and others as unnatural.

Efforts are being made globally to gain a deeper insight into women’s motivations for reconstruction. Survey-based studies in high-income countries, such as the US and France, found that women who pursue breast reconstruction may be influenced by the desire to feel attractive, feel whole again, and improve self-confidence.^34,35^ Qualitative work from Sweden suggested that women who opted out of reconstruction were motivated by prioritizing healing and avoiding the additional burden associated with surgery.^26^ The literature is lacking studies exploring women’s reasons for reconstructive choices in low- and middle-income countries. One survey-based study from India found that of women who did not pursue reconstruction, 75% cited that it was because they felt it was unnecessary or not a priority to them.^36^ Our study adds a qualitative perspective to the sparse literature on women’s attitudes and opinions toward breast reconstruction in low- and middle-income countries. Body image concerns and healing priorities appear universal across countries. The sub-Saharan African women interviewed in this study placed a specific emphasis on whether breast reconstruction could restore functionality or be beneficial to their healing, underscoring the importance of discussing how reconstruction affects breast function, including breastfeeding expectations.

Few studies have qualitatively explored the role life stage plays in women’s views of breast reconstruction. In a qualitative research study of Asian immigrant women in the US, participants reported that breasts became unnecessary once a woman had secured a partner and fulfilled her roles as wife and mother.^27^ Additionally, a survey of French women older than 65 years found that those declining reconstruction were more likely to consider themselves too old compared with those pursuing it (66% vs 4%), despite all participants being older than 65 years.^34^ Our findings align with these studies and expand our understanding of the influence of life stage by offering insights on this topic from women not represented in this literature. Contrary to assumptions, a US and Canadian study found that older women (aged ≥60 years) reported better sexual, physical, and psychosocial outcomes than younger women (aged <45 years) 2 years after delayed breast reconstruction.^37^ These findings suggest that age-specific counseling should be provided to older women, allowing them to make informed decisions on breast reconstruction that align with their treatment goals, potentially benefiting older women in sub-Saharan Africa similarly to those in the US and Canada.

Finally, the role of religion and spirituality in health care decision-making in this context should not be overlooked. Within communities in sub-Saharan Africa, there are accepted beliefs among patients that cancer is caused by a higher power, such as ancestors or God.^38,39^ Consequently, many women in sub-Saharan Africa initially turn to alternative treatments, such as herbal medicine or prayer camps, and often seek hospital care only when their breast cancer symptoms have become severe.^40,41,42^ In our study population, many women had accepted mastectomy as part of God’s plan. Paradoxically, while accepting breast removal, they viewed breast re-creation as contradicting their religious beliefs. These findings suggest that physicians should help patients to navigate cultural, religious, and spiritual considerations specific to reconstruction, even after they have accepted mastectomy, ensuring that care aligns with patients’ core values and beliefs. Additionally, incorporating discussions about reconstruction alongside with, or shortly after, discussions about mastectomy might allow it to be framed as part of the overall treatment process, potentially influencing the way reconstruction is perceived.

### Limitations

Our study should be viewed in the context of limitations. Interview dynamics and translation may have influenced data quality despite mitigation efforts such as private rooms, female interviewers, and frequent postinterview meetings to discuss clarifications with the local team. Furthermore, we did not formally track reasons why patients declined to participate, which represents important information that may help determine whether our study design biased our sample in ways affecting our ability to address our aim. Additionally, with our team being positioned on a different continent, we could not obtain participant validation of findings, though we attempted to mitigate this limitation through validation from our local collaborators’ perspectives.

## Conclusions

In this qualitative study of patients with breast cancer in Ghana and Ethiopia, we found that women’s opinions toward breast reconstruction were shaped by self-identity, breast functionality, life stage, and religious beliefs. By taking a more nuanced approach, these findings provide essential insights on patient priorities that should be considered in the design and delivery of culturally appropriate information to sub-Saharan women about reconstruction. The goal is to facilitate informed decisions about holistic breast cancer care, which may improve patient care and overall quality of life.

## Notes

Supplement 1. **eMethods.** Final Iteration of the Interview Guide **eTable.** Codebook

Supplement 2.
Data Sharing Statement
