# Supplementary material for: Patients’ Perspectives on Breast Reconstruction in Sub-Saharan Africa
Source: JAMA Netw Open. 2025 Jun 26;8(6):e2517749. doi: 10.1001/jamanetworkopen.2025.17749 (PMC12203280; doi:10.1001/jamanetworkopen.2025.17749)
Supplement: Supplement 1. — eMethods. Final Iteration of the Interview Guide eTable. Codebook [file jamanetwopen-e2517749-s001.pdf]

Supplemental Online Content

Guzman NM, Baglien BD, Kassa ES, et al. Patients' perspectives on breast reconstruction in sub-Saharan Africa. *JAMA Netw Open*. 2025;8(6):e2517749. doi:10.1001/jamanetworkopen.2025.17749

eMethods. Final Iteration of the Interview Guide

eTable. Codebook

This supplemental material has been provided by the authors to give readers additional information about their work.

© 2025 Guzman NM et al. *JAMA Network Open*.

eMethods. Final Iteration of the Interview Guide

AIM 1 Interview Instrument

SABR Study

University of Michigan Medicine

Introduction

Thank you for agreeing to speak with me, I am so grateful. I am very much interested in your experiences and feelings around breast cancer, mastectomy, and feelings you might have about body and body reconstruction. This is a qualitative interview, so I am hopeful that you will provide as much detail as possible—you can think of this as an opportunity to tell your story about what happened. Your stories are important and sharing what happened may shed light on how we can improve healthcare for other women. Some of these questions may seem too personal or intrusive—you do not have to answer any of these questions and can stop this interview at any time. There are no right or wrong answers to these questions, only your own experience. I will also read scenarios about a group of ladies who are facing certain situations and ask your opinion on their circumstances. Our goal is to improve the care when women learn they must have a mastectomy, or surgical removal of the breast. I am the only one who will know what you share in your interview, and this is confidential. Our goal is to improve the care that women receive and improve the quality of life for other women who have faced mastectomy. I am going to record this conversation but anything that you say that might identify you will be redacted, and I will not share this information with anyone outside of this study. Do you have any questions about me or this study?

Domain: Background

1. Tell me about yourself.
    a. Let's start with family. Tell me about your family. What is your home life like?
    b. Do you do work outside of the home? Like what? Tell me about what kind of work you do.
    c. What do you do for fun? Do you have any hobbies?

Domain: Breast Cancer Narrative

1. When you hear the word cancer, what do you think of?
2. What do you think causes cancer?
    a. What do you think caused your cancer?
    b. What did your health care provider tell you about the cause?

© 2025 Guzman NM et al. *JAMA Network Open*.

3. Starting the story anywhere you'd like and ending anywhere you'd like, tell me the story about how you learned you had breast cancer.
4. How did you feel when you were initially diagnosed?
5. What were your major concerns?

Domain: Disease and Treatment

1. Tell me about **what happened after** you learned you had breast cancer?
   a. Where did you get information about your diagnosis? (internet, other people?)
   b. What treatment options did you consider? What kind of treatments were you offered? What about surgery? What about chemo? What about radiation? Did you know about any other options?
   c. What treatment options did you receive? How did you decide?
2. Tell me about how you learned you would undergo the surgical removal of your breast?
   a. Talk to me about how you thought through the impact of this surgery on your overall health.
   b. How did you talk to your family about the surgery to remove your breast?
      i. What about friends or other people?
3. From when you learned that you would need surgery to remove your breast, how long was it before you actually had one? What happened in that time?
   a. (If the participant waited): What was happening that caused you to wait?
   b. what were your feelings about having the surgery? Were you scared or nervous?
   c. How did you manage those fears?
4. Tell me about the experience of having surgery to remove your breast.
   a. What was the surgery like?
   b. How did you think about your body?
   c. How did your husband react? What about the rest of your family?
   d. How do you think not having breasts changed the way people perceive you?

Domain: Loss of breast(s):

1. What does it mean to you to lose one or both breasts?
   a. Did this change how you felt as a woman?
2. How did you talk to your family or husband about this?
3. What about work? Did this affect work at all?
4. What did you think about your changed body?
5. Do you miss having breasts?
   a. Did anything positive come about after your mastectomy?

© 2025 Guzman NM et al. *JAMA Network Open*.

Domain: Quality of life and Well-Being

1. What does it mean to have quality of life?
2. How about well-being? What does it mean to say “good well-being”?
3. How would you say your quality of life was before cancer?
   a. How about after?
   b. Talk to me about some of the positive things that have happened since cancer.
   c. Did you ever feel downhearted or blue after your surgery to remove your breast?
      i. If yes, what do you think contributed to your feelings?

Domain: Reconstruction and Knowledge

1. Have you ever heard about women who have any reconstructive (plastic surgery) surgery after undergoing surgery to remove one or both breasts? If participant has not heard of this procedure read the following.

Breast reconstructions are operations that restore the form and appearance of the breast after surgery to remove one or both breasts for breast cancer treatment.

   a. What do you think about that/What is your opinion about breast reconstruction?
   b. What do you think/feel about breast reconstruction?
2. Did anyone ever speak with you about your options with breast reconstruction? Tell me about that.
3. If you did not receive breast reconstruction, tell me about why you did not.
   a. Probes are tailored in response to question—probe to understand barriers here.
4. If breast reconstruction were an option, would you consider such a thing? Tell me about what you would consider.
   a. Why would you want breast reconstruction?
   b. How would you pay for that?
5. How do you like to learn about health options?
   a. How would you like to learn about post-mastectomy reconstruction options?

What do you hope for other women who are facing mastectomy in Ghana/Ethiopia? What advice would you give them?

Is there anything about your experience that these questions did not cover?

Are there any questions that we should have asked that we didn’t?

© 2025 Guzman NM et al. *JAMA Network Open*.

eTable. Codebook

**Code**	**Definitions and Merging History**
Family	Any reference to family
Children	Merged with code Family\Daughter (Any reference to daughter); Merged with code Family\Son (Any reference to son
Husband	Any reference to husband
Other family/close friends	Any reference to other family or close friends
Support	Individuals who supported the participant or other ways they felt supported
Financial burden	Financial burdens like ceasing employment, struggle to pay, cutting back
Work	Any mention of work or financial survival
Financial sources	How or where individuals sourced their treatment financially
Hear Cancer	Answer to the question what do you think of when you hear the word cancer?
Death and survival	Discussion about being worried about dying or scared of dying or conversely survival discussions, "beating the disease" or doing something to prolong life.
Cancer Cause	Answer to the question what caused the cancer
Cancer Story	Answer to question: Tell me the story of how you learned you had cancer? Focus here on only the diagnosis, the rest of the cancer story should be coded with treatment.
Self-exams and self-care	Any reference to doing a self-exam or noticing any bodily changes
Treatment	The care pathway that women discuss, code the whole treatment story
Complications	Mention of complications associated with breast reconstruction
Chemo/Radiation	Mentions of chemo or radiation
Mastectomy	Any mention of mastectomy but always also code with treatment
Fear of medical care	Mentions of when women say they are scared of treatment of some sort
Pain	Any discussion of the physical manifestation of cancer or cancer treatment.
Hospital	Any reference to hospital

© 2025 Guzman NM et al. *JAMA Network Open*.

Age	Use when age is mentioned (ex. in context of qualifying feelings about breast reconstruction)
Care People	Any reference to care people
Doctor	Any reference to doctor, physician, surgeon
Someone else	Any reference to medical staff aside from doctor/physician/surgeon
Body/femininity/humanness	When women mentioned anything about their bodies that related to something about "womanness" like not feeling whole or not feeling human. (Merged with code Utility of breast)
Breastfeeding	Any reference to breastfeeding
Information	Where individuals got information from (Merged with code Advice\Received; Advice women received)
Media	Mentions of obtaining information from TV/Radio/social media
Other women/people	Mentions of obtaining information from other women or other people
Healthcare provider	Mentions of obtaining information from a healthcare provider
Social gossip or exclusion	Being socially isolated by others, others think cancer is communicable, or worried about neighbors gossiping
Mental Health	Discussion of mental or emotional state
Positive feelings about body	Mention of positive feelings about body
Coping/positive feelings	How women felt about their ability to keep living positively/negatively with cancer. Includes how women cope with cancer. By cope we mean strategies women used to cope.
Negative feelings about body	Mention of negative feelings about body
Neutral feelings about body	Mention of neutral feelings about body
Feeling sad	When women talk about "depression" but also sad or another term
QOL	Answer to the question what does quality of life mean to you
Well-being	Answer to the question of what does well-being mean to you
Breast Recon	Code full discussion about breast reconstruction
Feelings	Discussion of feelings related to breast reconstruction

© 2025 Guzman NM et al. *JAMA Network Open*.

Desire	Discussion of desire for breast recon, this can include the ability to pay for it.
Prosthetics	Mention of prosthetic breast
Hope for	Answer to question: What do you hope for (Merged with code Advice\Given)
Religion	Mentions of God, Jesus, any religion.

© 2025 Guzman NM et al. *JAMA Network Open*.
