# Supplementary material for: Patients’ Perspectives on Breast Reconstruction in Sub-Saharan Africa
Source: JAMA Netw Open. 2025 Jun 26;8(6):e2517749. doi: 10.1001/jamanetworkopen.2025.17749 (PMC12203280; doi:10.1001/jamanetworkopen.2025.17749)
Supplement: Supplement 2. — Data Sharing Statement [file jamanetwopen-e2517749-s002.pdf]

Data Sharing Statement

Guzman. Patients' Perspectives on Breast Reconstruction in Sub-Saharan Africa: A Qualitative Study. *JAMA Netw Open*. Published June 26, 2025.
doi:10.1001/jamanetworkopen.2025.17749

Data
Data available: No

Additional Information
Explanation for why data not available: This qualitative data will be used for our future mixed-methods study.
